# Genome-Wide Analysis of the *PYL* Gene Family and Its Expression Dynamics in Response to Abscisic Acid in Tomato

**DOI:** 10.3390/ijms27177737

**Published:** 2026-08-29

**Authors:** Nazia Jan, Aoyu Yang, Tongyun Sha, Zhangping Li, Ji Sun, Jinghua Yang, Rana Muhammad Amir Gulzar

**Affiliations:** 1 Laboratory of Germplasm Innovation and Molecular Breeding, Institute of Vegetable Science, Zhejiang University, Hangzhou 310058, China; 11816120@zju.edu.cn (N.J.); 22416058@zju.edu.cn (A.Y.); 11916064@zju.edu.cn (T.S.); lzp314@zju.edu.cn (Z.L.); yangjinghua@zju.edu.cn (J.Y.); 2 Southern Zhejiang Key Laboratory of Crop Breeding, Wenzhou Academy of Agricultural Sciences, Wenzhou 325006, China; sunji@wzvcst.edu.cn; 3 Hainan Institute, Zhejiang University, Yazhou Bay Science and Technology City, Sanya 572025, China

**Keywords:** ABA, *Pyrabactin resistance 1-like* (*PYL*) family, phylogenetic, gene expression, tomatoes

## Abstract

The plant hormone abscisic acid (ABA) plays a crucial role throughout the plant life cycle and in adaptive responses to environmental stresses. The pyrabactin resistance 1-like (PYR/PYL/RCAR) proteins act as key regulators in the ABA signal transduction pathway by functioning as direct receptors for ABA. Although *PYL* genes have been identified in a variety of plant species, their evolutionary and structural characteristics in tomatoes (*Solanum lycopersicum*) remain elusive. To address this gap, we identified nine *SlPYL* genes, which were classified into three subfamilies: I (two genes), II (three genes), and III (four genes), and their encoded proteins were predicted to be primarily localized in the cytosol and chloroplast. Structural analysis revealed diverse exon–intron organizations along with five conserved motifs. All identified *SlPYLs* contained the START domain (PF10604), validating their identity as actual PYL proteins. Prediction of *cis*-acting regulatory elements in *SlPYL’s* promoter regions was found to be associated with light responsiveness, hormone signaling, stress responses, and plant growth and development. Prediction of post-translational modification sites indicated that *SlPYLs* are predominantly phosphorylated and acetylated at serine and lysine residues, respectively. Tertiary structure modeling demonstrated conserved three-dimensional architectures among SlPYL proteins, supporting their functional conservation. Expression profiling revealed that specific *SlPYL* genes exhibit distinct expression patterns across different tissues (root, leaf, and bud) following ABA treatment, indicating functional diversification. Considering the well-established negative correlation between ABA accumulation and bud outgrowth, the ABA-induced differential expression (3~5-fold) of some *SlPYL* genes (*SlPYL3*, *SlPYL4*, *SlPYL7*, and *SlPYL8*), particularly in bud tissues after 24 hpt, suggests a potential role in ABA-mediated suppression of bud outgrowth. However, these functional inferences are primarily based on genome-wide computational analyses and expression profiling and therefore require further experimental validation.

## 1. Introduction

A tomato (*Solanum lycopersicum*) is an economically important vegetable crop and a widely used model system for studying floral development and fruit biology in sympodial plants [1]. Plant architecture is a critical determinant of crop productivity, and traits such as branching patterns, flower number, and fruit yield are tightly regulated by hormonal signaling networks [2]. Phytohormone- and nutrient-induced changes play a promising role among the various biochemical and physiological processes required to promote plant growth and development [3,4]. Throughout their life cycle, plants are also exposed to a wide range of adverse conditions that can be broadly classified as biotic and abiotic stresses [5,6]. Biotic stresses include pathogens and herbivores, whereas abiotic stresses comprise drought, salinity, temperature extremes, and nutrient imbalance [7,8].

Recent studies further indicate that ABA signaling interacts with ROS and other stress-responsive pathways, thereby integrating environmental signals with plant growth and developmental responses. Under salinity, drought, and temperature stress, excessive ROS accumulation can cause oxidative damage to cellular membranes and macromolecules, whereas antioxidant enzymes such as superoxide dismutase (SOD), peroxidase (POD), and catalase (CAT) contribute to ROS detoxification and maintenance of cellular redox homeostasis. Functional characterization of *FvNAC29* and *VhMYB15* has demonstrated that enhanced tolerance to salt, cold, and drought stress can be associated with increased SOD, POD, and CAT activities and reduced accumulation of H_2_O_2_ and malondialdehyde (MDA), highlighting the importance of ROS-scavenging mechanisms in transcriptionally regulated stress adaptation [9,10].

Among these regulatory molecules, abscisic acid (ABA) is a central phytohormone that coordinates plant responses to environmental stress while also regulating normal growth and development. Evidence from functional studies indicates that ABA-responsive transcription factors can contribute to drought and salinity tolerance by activating stress-responsive pathways and enhancing antioxidant capacity. For example, *MbWRKY3* expression was induced by drought, salinity, and exogenous ABA, and its overexpression in tobacco enhanced drought tolerance, accompanied by increased SOD, CAT, and POD activities and reduced H_2_O_2_ and MDA accumulation [11]. Similarly, *MbWRKY50* enhanced drought and cold tolerance in a transgenic tomato, with increased antioxidant activity and reduced ROS accumulation, while also affecting ABA-associated stress-responsive genes [12].

Abscisic acid (ABA) has also emerged as an important regulator of shoot branching. Previous studies have shown that ABA negatively regulates axillary bud outgrowth and contributes to the maintenance of bud dormancy in *A. thaliana*, suggesting that ABA signaling plays an important role in controlling branching and overall plant architecture [13,14]. In particular, ABA has been reported to negatively regulate bud outgrowth, suggesting a potential role for ABA signaling components in shaping plant architecture [13].

Pyrabactin resistance 1-like proteins (PYLs), members of the START domain-containing ligand-binding protein family, possess hydrophobic cavities that enable direct binding to ABA [15]. These proteins function as core components of the ABA signaling pathway, which also includes protein phosphatase 2C (PP2C) as a negative regulator [16] and SNF1-related protein kinase 2 (SnRK2) as a positive regulator [17]. Under low ABA conditions, PP2C interacts with SnRK2 and suppresses its activity through dephosphorylation, thereby maintaining the ABA signaling pathway in an inactive state [18]. In contrast, elevated ABA levels promote ABA binding to PYL receptors, which inhibits PP2C activity and subsequently activates SnRK2-mediated downstream signaling [19].

Previous studies have advanced our understanding of tomato *PYL* genes through genome-wide identification, evolutionary analysis, and functional characterization of their roles in ABA perception and stress responses [20,21]. However, these studies have mainly focused on ABA-mediated abiotic stress tolerance, while the involvement of *SlPYLs* in regulating tomato plant architecture, particularly axillary bud development, remains poorly understood. Except in tomatoes, genome-wide identification and experimental validation of *PYL* genes have also been reported in several other plant species, including 13 *PYL* genes in rice [22], 12 in maize [23], 14 in cucumber [24], nine in *Brachypodium distachyon* [25], 25 in oilseed rape [15] and 27 in soybean [26].

The *PYL* gene family has been widely studied in different plant species due to its essential role in ABA signaling. Functional studies have demonstrated that overexpression of *OsPYL5*, *OsPYL8*, and *OsPYL9* in rice [22], *ZmPYL3*, *ZmPYL9*, *ZmPYL10*, and *ZmPYL13* in maize (*Zea mays*) [23], and/or *AtPYL13* in *Arabidopsis* enhances plant sensitivity to ABA [27]. Moreover, exogenous ABA inhibits axillary bud outgrowth, while loss-of-function mutants in ABA signaling (*aba1-1*, *abi1-1*) display enhanced bud activation. ABA restricts lower bud outgrowth and promotes correlative inhibition under both high and low R:FR light. Although not entirely dormant, the fate of lower buds is plastic, and their growth and development can be retarded or accelerated by a number of factors [14].

In the present study, a comprehensive genome-wide analysis of the *PYL* gene family in tomatoes was conducted. In this study, the *PYL* gene family was systematically investigated in the tomato cultivar “Alisa Craig”, a widely used genetic background for studying tomato development and hormonal regulation. By combining genome-wide characterization with tissue-specific and ABA-responsive expression analyses in axillary buds, this study provides new insights into the potential roles of *SlPYL* genes in ABA-mediated regulation of tomato architecture. A total of nine *SlPYL* genes were identified and systematically characterized based on their abundance in bioinformatic analyses, including phylogenetic relationships, gene structures, conserved domains and motifs, chromosomal distribution, promoter *cis*-regulatory elements, three-dimensional protein structures, and predicted post-translational modification sites on different amino acid residues. Furthermore, expression profiling of *SlPYLs* under ABA treatment across different tissues (root, leaf, and bud) was performed to investigate their potential functional roles in distinct plant parts. Moreover, expression analysis of *SlPYLs* following ABA application in the buds of tomato plants at different time frames indicated their importance in axillary bud growth. These findings provide a foundation for understanding the involvement of *SlPYL* genes in ABA-mediated signaling and their possible contribution to the regulation of the plant architecture of tomatoes.

## 2. Results

### 2.1. Identification and Physicochemical Properties of PYL Gene Family in Solanum lycopersicum

**Figure 1:**
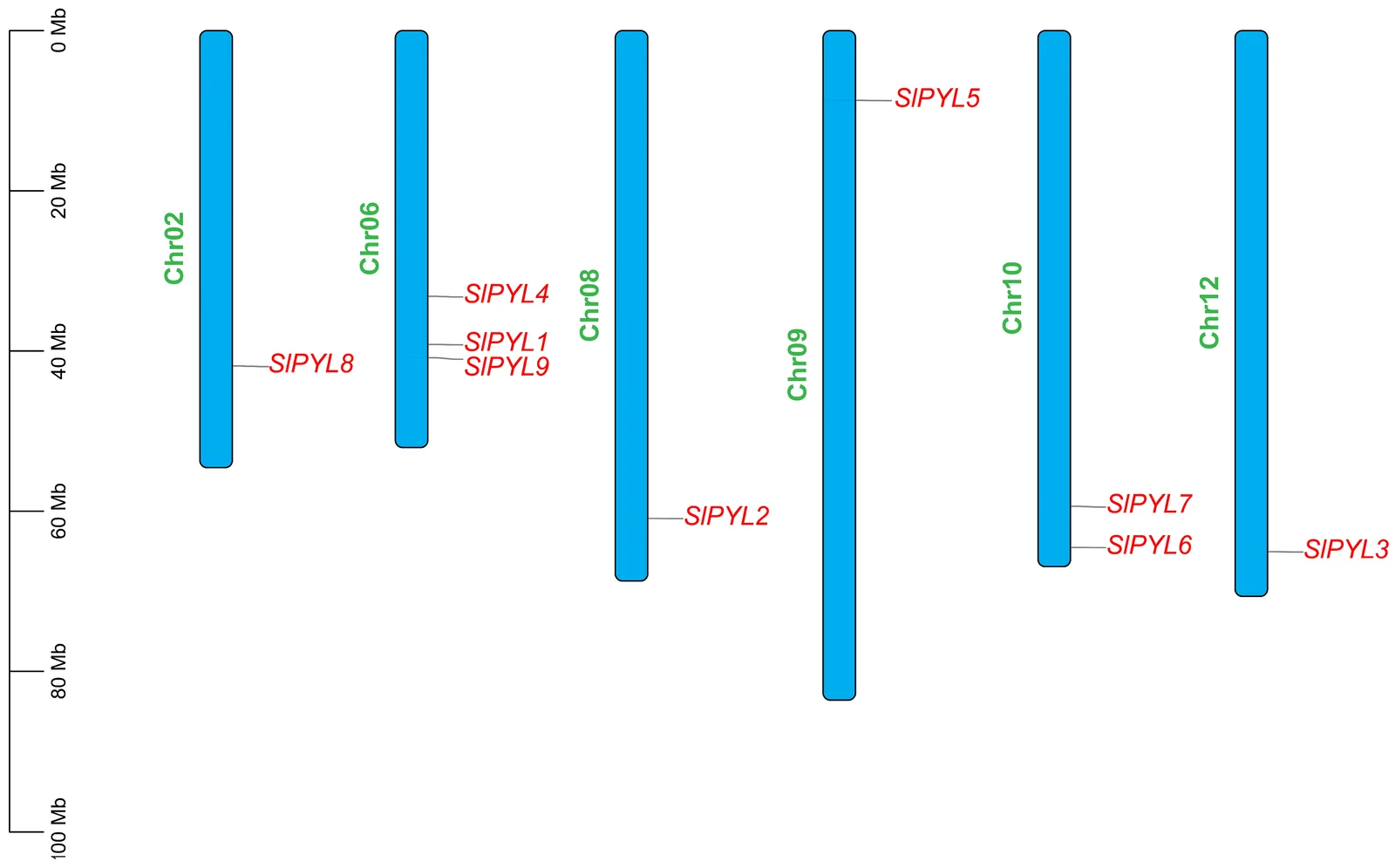
Chromosomal localization of *SlPYL* genes in *S. lycopersicum*. *SlPYL* genes are mapped onto their respective chromosomes, with gene positions indicated by black horizontal lines and gene names shown in red.

To identify PYL proteins in *S. lycopersicum*, thirteen known PYL sequences from *A. thaliana* were retrieved from the TAIR database and used as queries in BLASTP searches against the tomato genome assembly (SL4.0). A total of nine candidate *SlPYL* proteins were identified and named based on their sequence similarity to the corresponding AtPYL proteins (Table 1). The predicted SlPYL proteins varied in length from 190 to 322 amino acids. All identified SlPYLs contained the conserved START (PF10604) domain along with the characteristic SGLP gate motif, confirming their identity as ABA receptors. The theoretical isoelectric points (pI) ranged from 5.03 to 6.65, indicating variation in their charge properties. Chromosomal mapping revealed that *SlPYL* genes are distributed across chromosomes 02 to 12. Specifically, *SlPYL8* is located on chromosome 02; *SlPYL1*, *SlPYL4*, and *SlPYL9* on chromosome 06; *SlPYL2* on chromosome 08; *SlPYL5* on chromosome 09; *SlPYL6* and *SlPYL7* on chromosome 10; and *SlPYL3* on chromosome 12 (Figure 1; Table 1). A relatively higher number of genes were observed on chromosome 06, suggesting possible local gene duplication or retention events. Overall, the distribution pattern indicates that *SlPYL* genes are widely dispersed across the tomato genome.

### 2.2. Phylogenetic Analysis of SlPYLs and Related PYL Proteins

**Figure 2:**
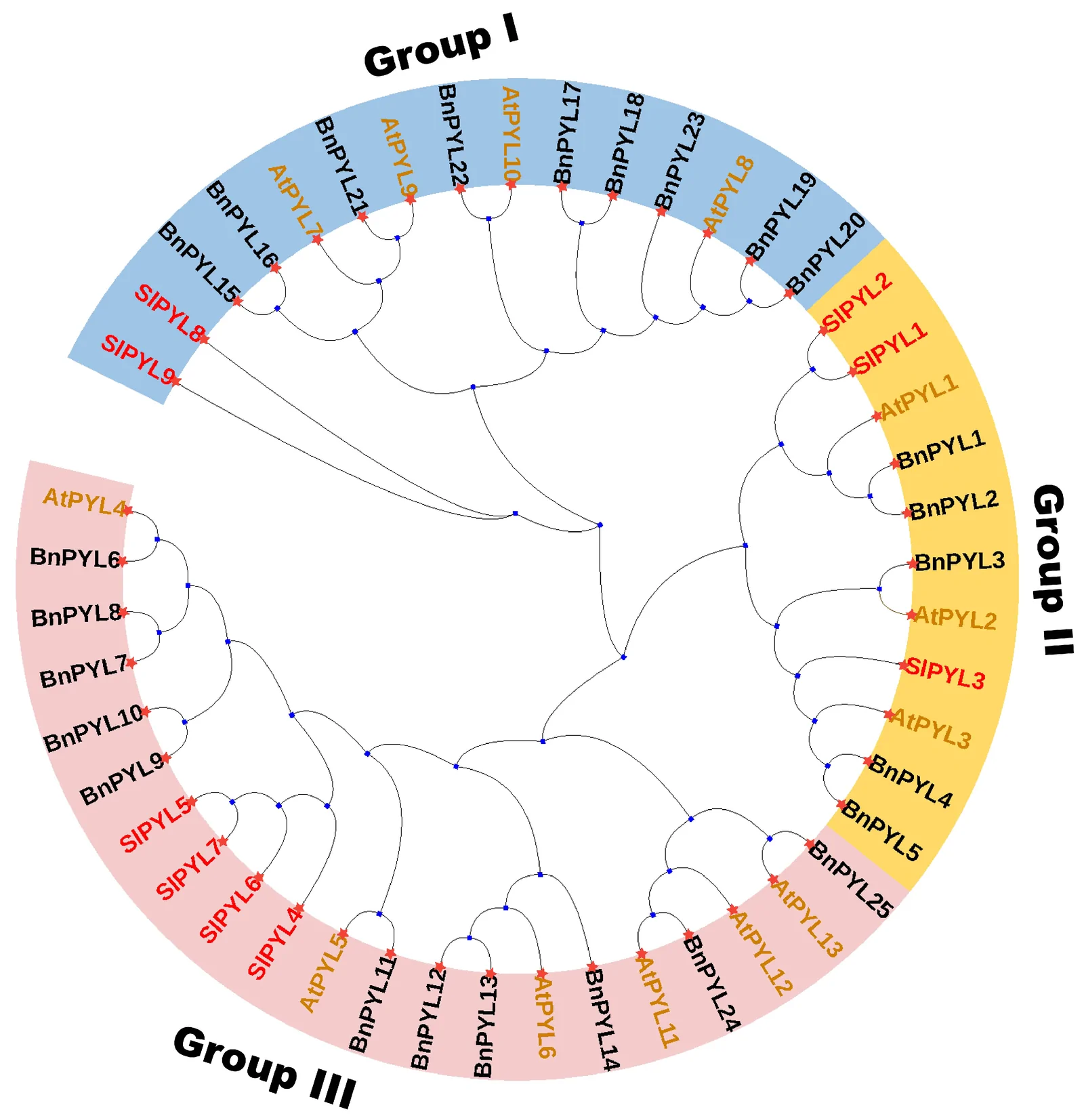
Phylogenetic analysis of PYL proteins from *Arabidopsis thaliana*, *Brassica napus*, and *S. lycopersicum*. A maximum likelihood (ML) phylogenetic tree was constructed using MEGA X based on full-length AtPYL, BnPYL, and SlPYL protein sequences with 1000 bootstrap replicates and further visualized using the iTOL online tool. The PYL proteins were classified into three major groups (I, II, and III). Different label colors represent PYL proteins from *A. thaliana*, *B. napus*, and *S. lycopersicum*. Internal nodes are indicated by blue circles while red asterisks indicated respective leaf nodes. Group I contained 1 AtPYL, 4 BnPYLs, and 2 SlPYLs; Group II contained 3 AtPYLs, 5 BnPYLs, and 4 SlPYLs; and Group III contained 6 AtPYLs, 11 BnPYLs, and 3 SlPYLs.

To investigate the evolutionary relationships of the *PYL* gene family, a maximum likelihood (ML) phylogenetic tree was constructed using full-length PYL protein sequences from *A. thaliana* (13), *B. napus* (25), and *S. lycopersicum* (9) (Figure 2). The phylogenetic tree classified the PYL proteins into three distinct groups (I, II, and II), consistent with previous studies. The analysis revealed that SlPYL proteins clustered closely with their homologs from *A. thaliana* and *B. napus*, indicating a high degree of evolutionary conservation across species. However, variations in gene number and distribution among the three species suggest lineage-specific expansion and diversification of the *PYL* gene family. Interestingly, the clade associated with AtPYL12 lacked corresponding orthologs from both *B. napus* and *S. lycopersicum*, which may indicate gene loss or functional divergence during evolution. Overall, these findings suggest that the *SlPYL* gene family has undergone diversification, potentially driven by gene duplication events and subsequent evolutionary adaptation.

To investigate the evolutionary relationships of tomato PYL proteins, a phylogenetic tree was constructed using PYL sequences from tomatoes, *Arabidopsis thaliana*, and *Brassica napus*. These species were selected as representative dicot plants with well-characterized genomes and conserved ABA signaling components. The phylogenetic analysis classified PYL proteins into three major groups (Groups I–III) as previously described in studies [15]. SlPYL members were distributed among all three groups, indicating evolutionary conservation as well as diversification of the PYL family. Proteins within the same group exhibited similar sequence characteristics and conserved motif compositions, suggesting potential functional similarities, whereas divergence among groups may reflect functional specialization during evolution.

### 2.3. Conserved Domain, Motif and Gene Structure Analysis of SlPYLs

**Figure 3:**
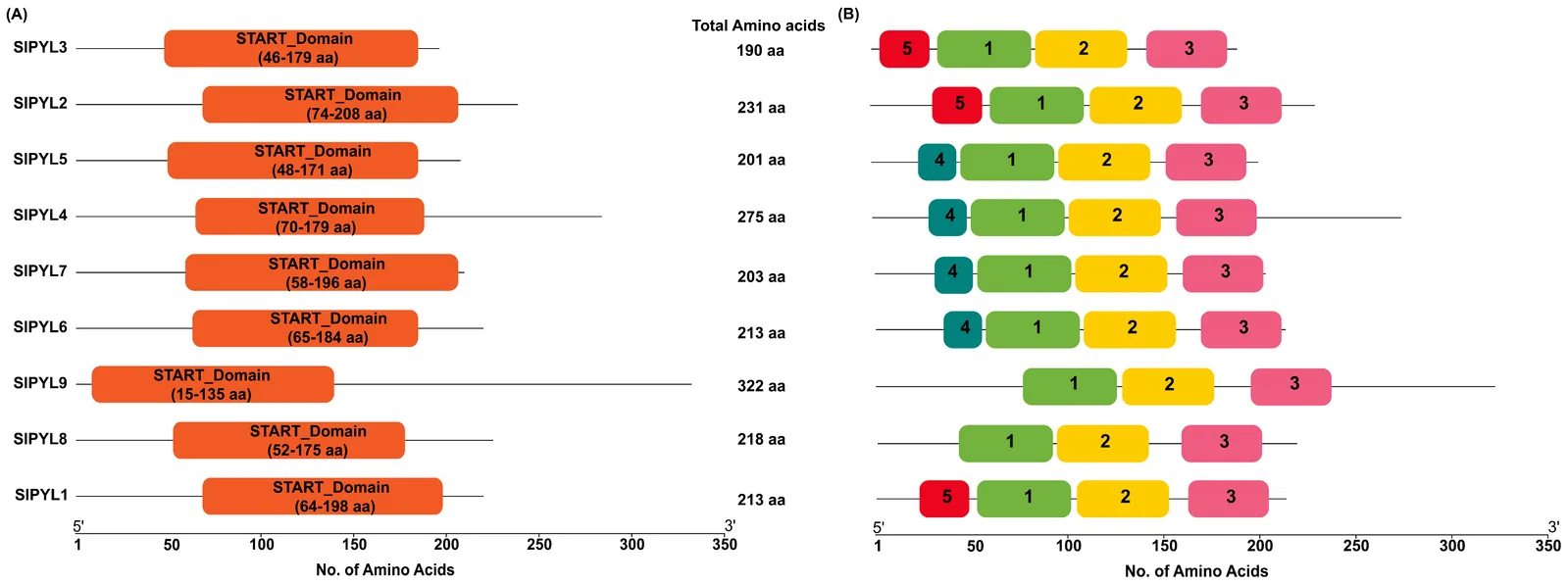
Conserved domain and motif architecture of SlPYLs proteins in *S. lycopersicum*. (**A**) Conserved domain organization of SlPYL proteins identified using NCBI-CDD. (**B**) Motif composition of SlPYL proteins, showing the distribution and position of conserved motifs along the protein sequences. Different colors represent distinct motifs identified using the MEME suite.

To identify the conserved functional domains in SlPYLs proteins, domain prediction analysis was performed using Pfam and NCBI conserved domain database (CDD). Domain composition analysis revealed that all SlPYLs contain the conserved START (PYL) domain (accession: PF10604), confirming their identity as members of the PYL family and supporting their conserved role in ABA perception and signaling (Figure 3A). However, the specific functional implications of this domain in downstream regulatory processes require further experimental validation.

To further explore conserved sequence features, all SlPYL protein sequences were analyzed using the MEME suite, which identified a total of five conserved motifs (Figure 3B). These motifs varied in length, ranging from 21 amino acids (motif 4) to 50 amino acids (motif 1). Group I members (SlPYL8 and SlPYL9) each contained three motifs. Group II members (SlPYL1, SlPYL2, and SlPYL3) each contained four motifs (1, 2, 3, and 5). Group III members (SlPYL4, SlPYL5, SlPYL6, and SlPYL7) each contained four motifs, including motifs 1, 2, 3, and 4. Overall, motif composition was highly conserved within each group, while variations among groups may reflect functional diversification of SlPYL proteins. The sequence logos and consensus amino acid compositions of the identified motifs are presented in Figure S1. Notably, all SlPYLs contained motif 2, which corresponds to the characteristic SGLP gate motif.

**Figure 4:**
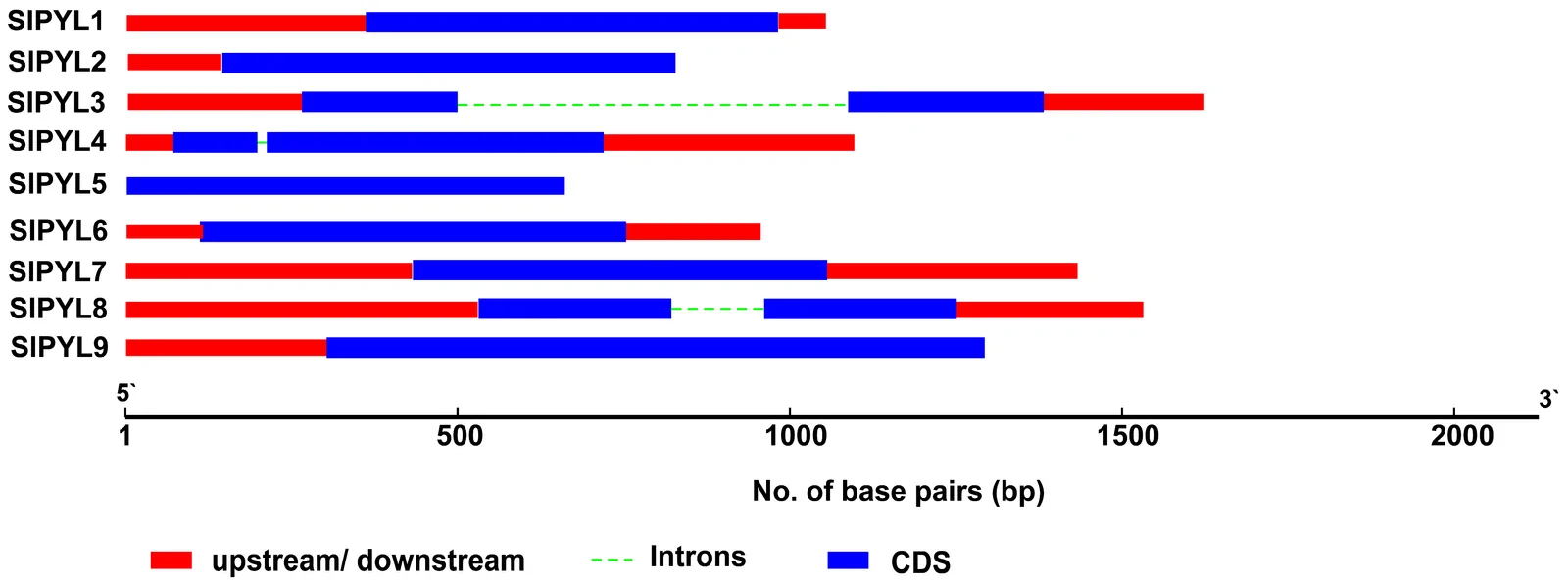
Gene structure organization of *SlPYL* genes in *S. lycopersicum*. Exons are represented by blue boxes, introns by dashed green lines, and upstream/downstream regions by red boxes. The lengths of genes are shown according to the genomic scale (bp).

Gene structure analysis revealed clear variation in exon–intron organization among *SlPYL* genes (Figure 4). Most *SlPYL* genes exhibited relatively simple gene structures, with several members lacking introns or containing only a single intron. Specifically, *SlPYL8* (Group I), *SlPYL3* (Group II), and *SlPYL4* (Group III) each contained a single intron, whereas the remaining *SlPYL* genes were intron-less. These patterns indicate that exon–intron organization varies among *SlPYL* subfamilies, suggesting structural divergence that may be associated with functional specialization during the evolution of this gene family in *S. lycopersicum*.

### 2.4. Promoter Cis-Acting Element Analysis of SlPYLs

To investigate the potential regulatory functions of *SlPYL* genes, *cis*-acting regulatory elements (CREs) were analyzed within the 2 kb upstream promoter regions using the PlantCARE database. The promoter regions of *SlPYL* genes were enriched with a diverse set of CREs associated with light responsiveness, phytohormone signaling, stress responses, and other regulatory processes, suggesting their involvement in multiple physiological pathways.

Light-responsive elements, including MRE, Box 4, AE-box, GAP-box, ACE, and GATA-motif, were widely distributed across the promoters of *SlPYL* genes. Among these, Box 4 was the most abundant element (42 occurrences), with *SlPYL2* containing the highest number (nine), indicating a potential role of light-mediated regulation in *SlPYL* gene expression. Furthermore, phytohormone-responsive elements such as ARE, ABRE, ERE, TCA-element, CGTCA-motif, GARE-motif, TGACG-motif, TGA-element, and P-box were also predicted. Notably, ABA-responsive elements (ABREs) were highly prevalent, suggesting that *SlPYL* gene expression is strongly influenced by ABA-mediated signaling. In particular, *SlPYL8* contained the highest number of ABREs (five), whereas *SlPYL3*, *SlPYL5*, and *SlPYL6* each contained two ABREs. Furthermore, the presence of TGACG- and CGTCA-motifs indicates that *SlPYL* genes may also be involved in other hormone-related signaling pathways. Moreover, stress-responsive elements, including MBS, LTR, GT1-motif, STRE, TC-rich repeats, W-box, and WUN-motif, were also widely detected. Among these, STRE elements (21) were particularly abundant, especially in *SlPYL2*, *SlPYL3*, and *SlPYL7* (four each). Similarly, GT1-motifs (12) were enriched across multiple genes, with *SlPYL1*, *SlPYL2*, *SlPYL3*, *SlPYL5*, and *SlPYL7* each containing two copies. MBS elements (11) were also frequently observed, particularly in *SlPYL1* and *SlPYL7* (three each), suggesting potential involvement in stress-responsive transcriptional regulation.

**Figure 5:**
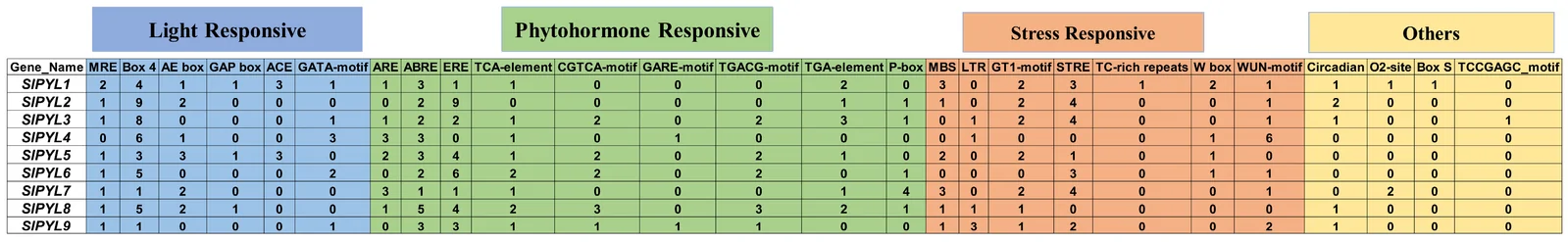
Distribution of *cis*-acting regulatory elements in the promoter regions of *SlPYL* genes in *S. lycopersicum*. *Cis*-elements were identified within the 2 kb upstream regions of *SlPYL* genes and classified into light-responsive, phytohormone-responsive, stress-responsive, and other regulatory categories. The number within each cell indicates the exact motif count. A value of “0” denotes the absence of that *cis*-element in the corresponding promoter.

In addition, other regulatory elements, including circadian, O_2_-site, Box S, and TCCGAGC motifs, were identified, indicating possible roles in broader aspects of plant growth and development. In contrast, circadian, O_2_-site, and Box S were relatively less abundant, suggesting limited contribution to specific regulatory pathways. Notably, the TCCGAGC motif, a predicted binding site for the PAT1 transcription factor, was identified in the promoter region of *SlPYL3* (Figure 5), suggesting potential transcriptional regulation by PAT1. Given that PAT1 has been implicated in regulating developmental processes, the presence of this motif in *SlPYL3* is particularly interesting and may indicate a previously unreported regulatory link between PAT1 and ABA receptor genes. Considering the relatively higher expression of *SlPYL3* in bud tissues and its strong induction under ABA treatment, this finding suggests that PAT1-mediated regulation of *SlPYL3* may contribute to ABA-dependent modulation of bud outgrowth. Overall, the diverse and combinatorial distribution of these *cis*-acting elements suggests that *SlPYL* genes are regulated through complex networks integrating light, hormonal, and stress-responsive signaling pathways, thereby contributing to plant growth and adaptive responses.

### 2.5. Homology Modeling and Structural Analysis of SlPYL Proteins

**Figure 6:**
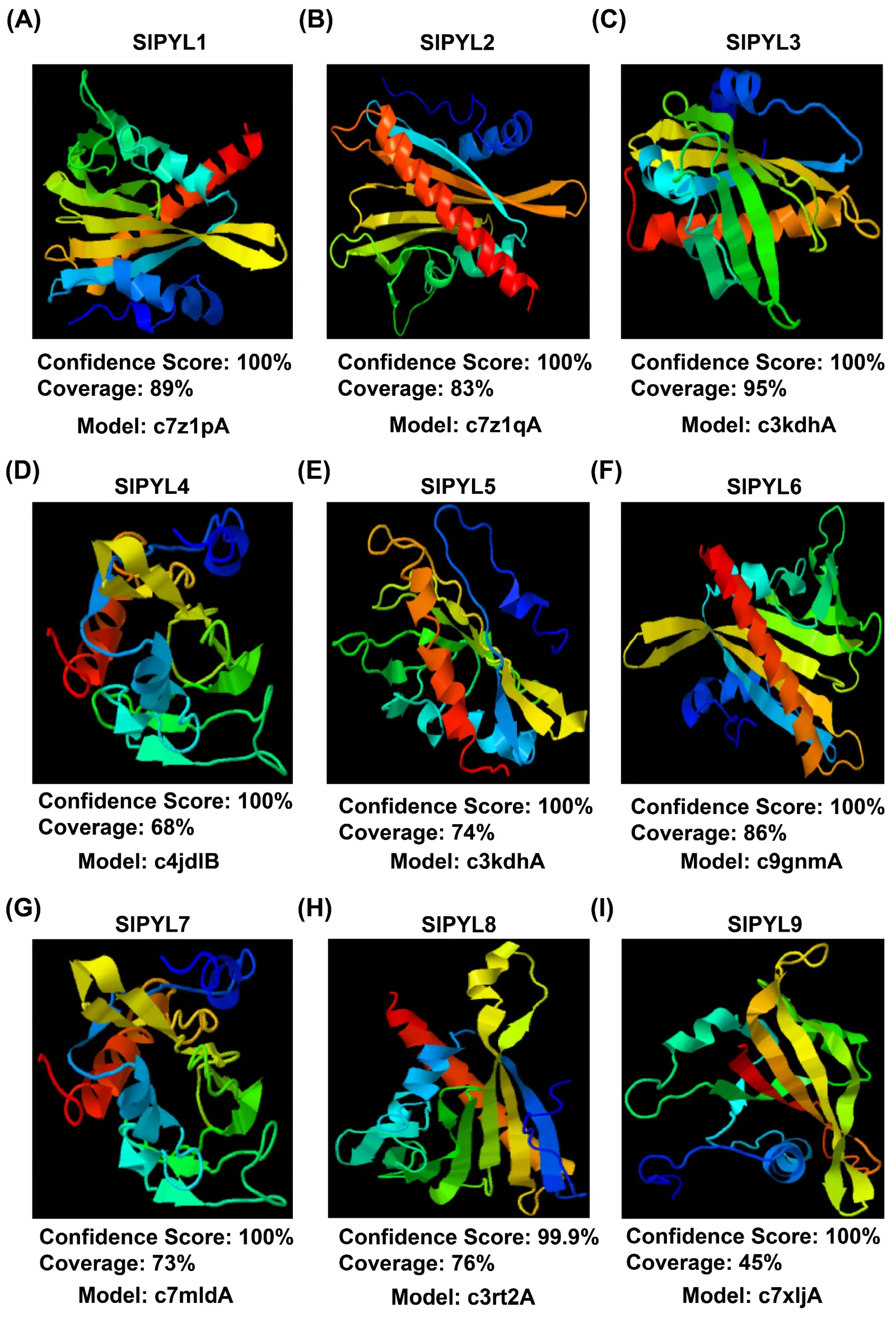
Predicted three-dimensional structures of SlPYL proteins generated using the Phyre2 server. (**A**–**I**) The models predicted for SlPYLs displayed in rainbow colors from the N-terminus to the C-terminus. The confidence score, alignment coverage, and template model information for each predicted structure are shown below the corresponding model.

Three-dimensional homology models of all identified SlPYL proteins were generated using the Phyre2 server (Figure 6). The predicted structures showed that all SlPYL proteins were modeled with very high confidence scores (99.9–100%), although the coverage values varied among members. The highest coverage was observed for SlPYL3 (95%), followed by SlPYL1 (89%) and SlPYL6 (86%), whereas the lowest coverage was recorded for SlPYL9 (45%). Analysis of the predicted secondary structures revealed that SlPYL proteins are predominantly composed of α-helices and β-strands, along with variable loop and disordered regions. In general, members of Group I (SlPYL8 and SlPYL9) exhibited relatively higher disordered content as compared to other group members (Table S1). Members from group II, SlPYL1, SlPYL2, and SlPYL3 contained 15%, 21%, and 19% alpha-helices (α-helices), respectively, and 34%, 34%, and 37% beta-strands (β-strands), respectively. Comparatively, SlPYL5 (group II) exhibited relatively higher proportions of β-strands (39%) (Table S1). These variations in secondary structural composition may reflect differences in protein flexibility and functional specialization among SlPYL proteins. The predicted tertiary structures further demonstrated that SlPYL proteins share a conserved overall folding pattern characteristic of the PYL family, despite variations in structural conformation and model coverage. Such structural conservation supports their common role in ABA perception, while the observed differences among individual members may contribute to functional diversification in *S. lycopersicum*.

### 2.6. Predicted Post-Translational Modification Sites in SlPYL Proteins

**Figure 7:**
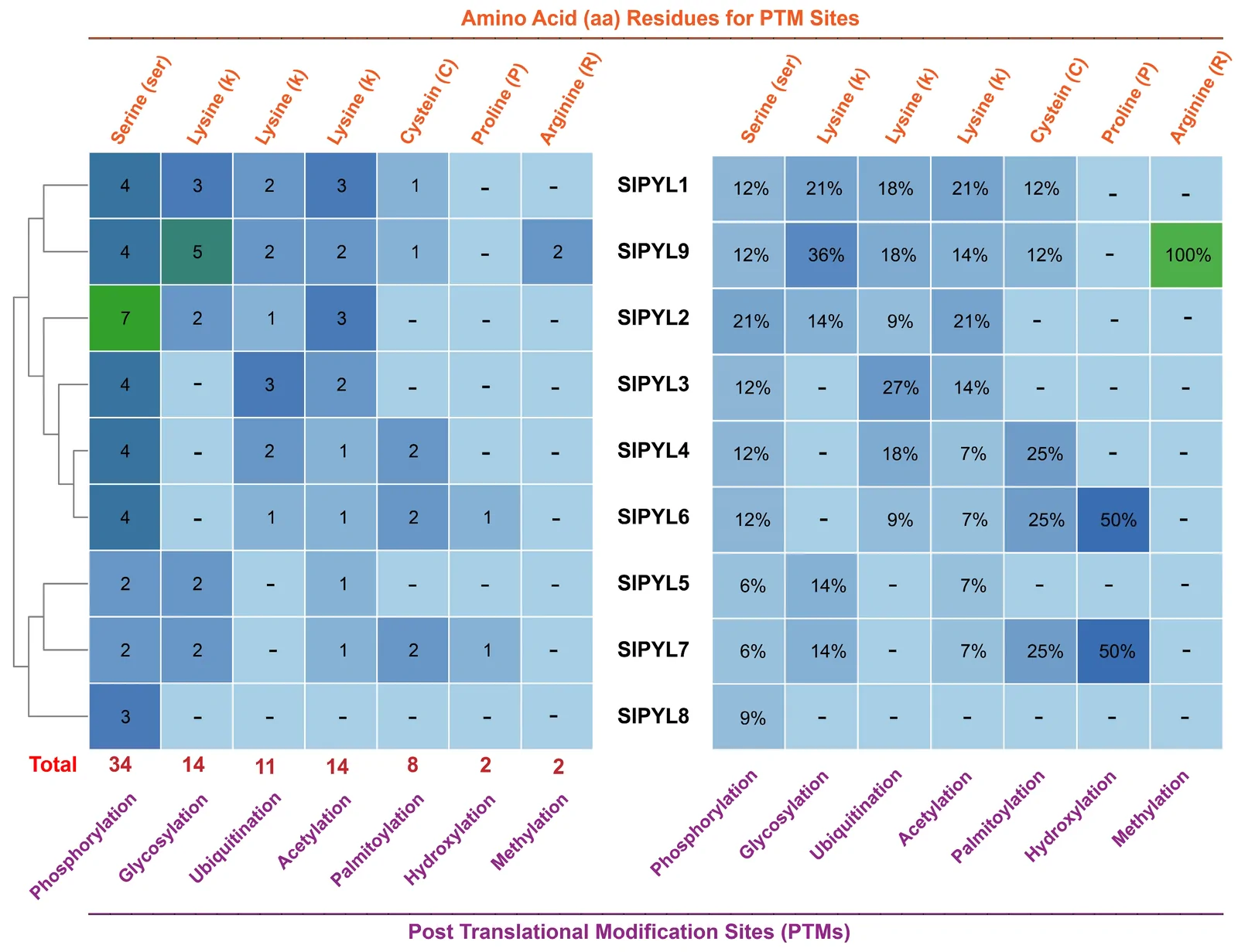
Predicted post-translational modification (PTM) sites in SlPYL proteins. The left panel shows the number of predicted PTM sites in each SlPYL protein according to amino acid residue type, whereas the right panel presents the corresponding percentage distribution. Predicted PTMs include phosphorylation, glycosylation, ubiquitination, acetylation, palmitoylation, hydroxylation, and methylation. The numbers at the bottom indicate the total predicted sites for each PTM category across all SlPYL proteins.

Potential post-translational modification (PTM) sites were predicted for all identified SlPYL proteins (Figure 7). Among the predicted PTMs, phosphorylation at serine residues was the most abundant modification. All SlPYL proteins contained at least one potential serine phosphorylation site. SlPYL2 exhibited the highest number of predicted serine phosphorylation sites (7), followed by SlPYL1, SlPYL3, SlPYL4, SlPYL6, and SlPYL9, each containing four such sites. These findings suggest that phosphorylation may represent a major regulatory mechanism affecting SlPYL protein function. In addition to phosphorylation, glycosylation sites were also predicted in several SlPYL proteins. These findings revealed that SlPYL9 contained the highest number of glycosylation sites (5), whereas some SlPYL members lacked any predicted glycosylation sites (Figure 7; Table S2). Overall, glycosylation was less frequent than phosphorylation, indicating that only selected SlPYL proteins may undergo this modification. Moreover, ubiquitination and acetylation at lysine residues were also detected in the SlPYL proteins. Acetylation sites were identified in most SlPYLs, with the majority of proteins containing at least one predicted acetylation site, while SlPYL8 lacked acetylation. Similarly, ubiquitination sites were predicted in several SlPYLs, indicating that these proteins may also be regulated through ubiquitin-mediated modification. Among the different PTM types, acetylation and ubiquitination were moderately represented compared with phosphorylation.

Additional PTMs, including palmitoylation, hydroxylation, and methylation, were predicted in a smaller number of SlPYL proteins. Palmitoylation sites at cysteine residues were identified in selected members, whereas hydroxylation at proline residues and methylation at arginine residues were the least frequent modifications (Table S2). Overall, these findings indicate that SlPYL proteins are particularly enriched in phosphorylation sites, while the presence of additional PTMs suggests multilayered post-translational regulation that may influence their biological functions in SlPYLs.

### 2.7. Expression Profiling of SlPYLs via qRT-PCR Analysis in Distinct Plant Parts and Post-Application of ABA

**Figure 8:**
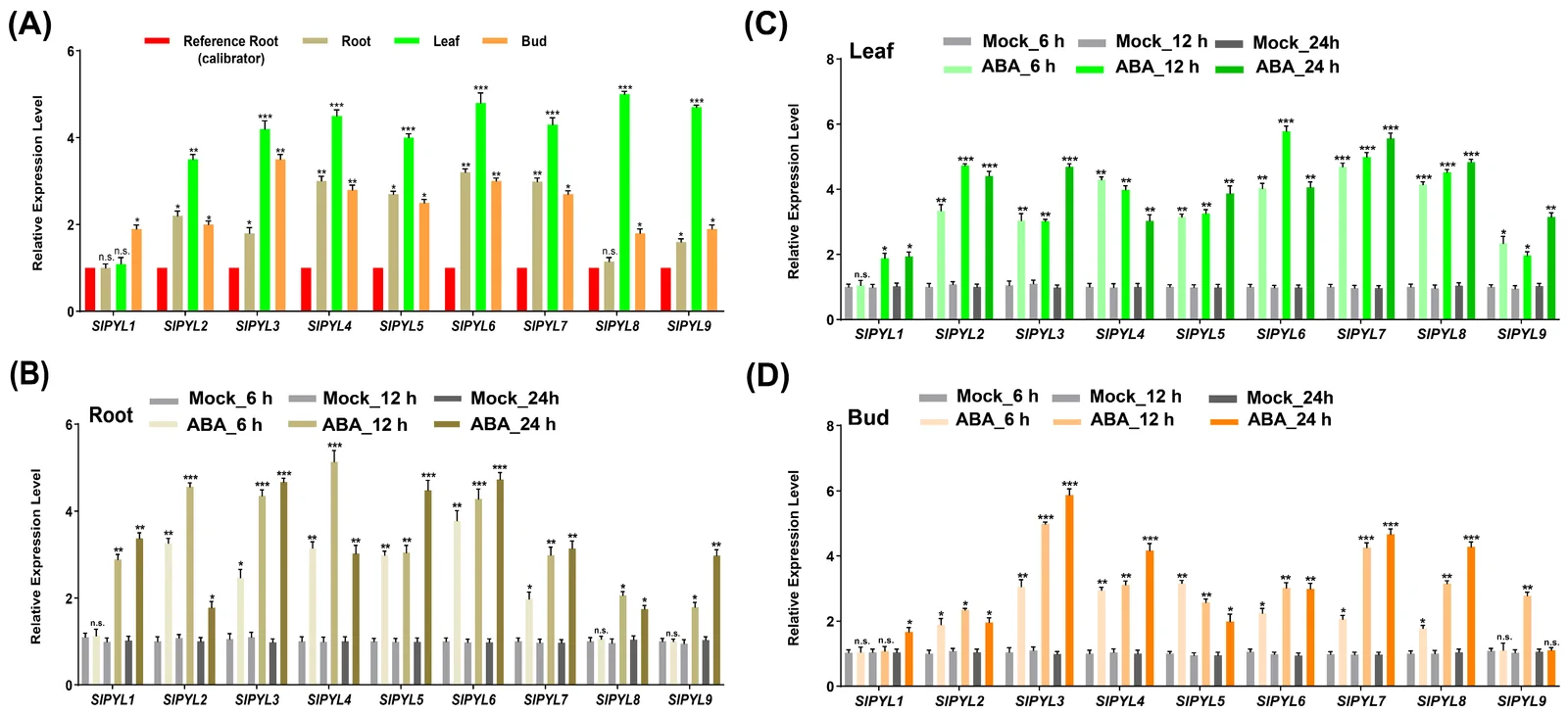
Relative expression profiling of *SlPYL* genes in different tissues and following ABA treatment. (**A**) Expression patterns of *SlPYL* genes in the reference root sample (calibrator), an independent experimental root sample, leaf, and bud tissues. The reference root sample (red bars) was used as the calibrator for relative quantification and assigned a relative expression value of 1. Relative transcript levels of the experimental root (olive bars), leaf, and bud samples were calculated using the 2^−ΔΔCt^ method. Statistical comparisons between the experimental root, leaf, or bud samples and the reference root were performed using replicate-level ΔCt values rather than the assigned calibrator value. (**B**–**D**) Expression patterns of *SlPYL* genes following exogenous ABA treatment in (**B**) root, (**C**) leaf, and (**D**) bud tissues at 6, 12, and 24 hpt. Relative transcript levels were calculated using the 2^−ΔΔCt^ method with *SlActin* (*Solyc03g078400*) as the internal reference gene. At each time point, the mean ΔCt value of the corresponding mock (water-treated) biological replicates was used as the reference for calculating ABA-responsive relative expression changes. Individual biological replicates were retained for estimation of biological variability, and data are presented as mean ± SD (*n* = 3 independent biological replicates). Statistical comparisons between ABA-treated and corresponding mock samples at each time point were performed using replicate-level ΔCt values and Student’s *t*-test. Asterisks indicate significant differences (*p* < 0.05, * *p* < 0.01, ** *p* < 0.001, *** *p* < 0.0001), whereas “n.s.” indicates no significant difference.

The expression patterns of *SlPYL* genes were analyzed using qRT-PCR across different plant tissues, including root, leaf, and bud (Figure 8A). Most *SlPYL* genes (*SlPYL2*, *SlPYL3*, *SlPYL4*, *SlPYL5*, *SlPYL6*, *SlPYL7*, and *SlPYL8*) exhibited detectable expression levels ranging from approximately 1.5~5-fold across the examined tissues. Among these, *SlPYL4*, *SlPYL5*, *SlPYL6*, and *SlPYL7* showed relatively higher expression levels in root tissues (approximately 2~3-fold) compared to other *SlPYLs*. In leaf tissues, most *SlPYL* genes displayed elevated expression levels, with the exception of *SlPYL1*, which showed comparatively lower expression. In bud tissues, *SlPYL3*, *SlPYL4*, *SlPYL6*, and *SlPYL7* exhibited relatively higher expression levels compared to other family members, with SlPYL3 showing the most prominent expression among them. Overall, the differential expression patterns of *SlPYL* genes across tissues suggest their involvement in multiple aspects of plant growth and development. Notably, the expression profiles revealed tissue-specific variation among the *SlPYL* genes, with several members exhibiting relatively high transcript abundance in leaves, while others were expressed in buds and additional tissues. These results suggest differential spatial expression patterns of *SlPYL* genes across tomato tissues.

To further investigate the regulatory response of *SlPYL* genes, their expression patterns were analyzed following exogenous ABA application at different time intervals (6, 12, and 24 hpt) (Figure 8B–D). The identified *SlPYL* genes contain ABA-responsive *cis*-elements in their promoter regions, supporting their potential responsiveness to ABA signaling. Overall, most *SlPYL* genes exhibited increased expression levels following ABA treatment, with more pronounced induction observed in leaf tissues compared to roots. In root tissues, these genes exhibited induction, although at relatively lower levels (3~3.5-fold) (Figure 8B). *SlPYL3* and *SlPYL7* showed the highest induction levels in leaves, reaching approximately ~5-fold and ~5.5-fold increases, respectively, at 24 hpt compared to water-treated control plants (Figure 8C). Similarly, in bud tissues, *SlPYL3* and *SlPYL7* showed significant ABA-induced expression, ranging from approximately 3~6-fold and 2.5~4.5-fold, respectively (Figure 8D). Notably, the expression levels of these genes increased progressively over time following ABA treatment. These findings are consistent with the enrichment of ABREs in *SlPYL* promoters, supporting their transcriptional regulation by ABA. Collectively, these results indicate that *SlPYL* genes are responsive to ABA signaling across different tissues. In particular, the enhanced and sustained expression of specific *SlPYL* genes in bud tissues suggests their potential involvement in ABA-mediated regulation of bud development and branching.

## 3. Discussion

The PYL protein family functions as key ABA receptors within the core PYR/PYL/RCAR-PP2C-SnRK2 signaling module, playing essential roles in regulating plant growth, development, and responses to abiotic stress [28,29,30]. *PYL* genes have been extensively identified and functionally characterized in several plant species, including potatoes, cucumbers, and rice [22,24,31]. However, their comprehensive characterization and potential functional roles in tomatoes, particularly in relation to plant architecture, remain less explored. The present study therefore extends current knowledge of the tomato ABA signaling system by integrating genome-wide characterization, structural and regulatory analyses, and tissue-specific and ABA-responsive expression profiling of the *SlPYL* gene family.

In this context, the present study provides a comprehensive genome-wide characterization of the *SlPYL* gene family in *S. lycopersicum*. A total of nine *SlPYL* genes were identified and classified into three subfamilies (I-III) (Figure 2), consistent with phylogenetic patterns reported in other plant species. The conservation of the START (polyketide_cyc2) domain across all *SlPYL* proteins confirms their identity as functional ABA receptors (Figure 3A). In addition, motif analysis revealed the presence of conserved sequence features, including the characteristic SGLP gate motif, which is known to be critical for ABA binding and receptor activity (Figure 3B). These findings are consistent with previous reports in other plant species [15,32], indicating strong structural conservation within the PYL family. Furthermore, predicted secondary and tertiary structural analyses demonstrated conserved folding patterns composed of α-helices, β-sheets, and loop regions, supporting the structural stability and functional conservation of SlPYL proteins. The conservation of these characteristic structural features suggests that the identified SlPYLs are likely to retain the basic molecular properties required for ABA perception, although biochemical and genetic experiments will be required to determine whether individual receptors differ in ABA affinity, ligand specificity, or downstream signaling activity.

In addition to phylogenetic relationships, the chromosomal distribution analysis revealed that *SlPYL* genes are unevenly distributed across the tomato genome, with chromosome 06 harboring a relatively higher number of family members. Such non-random genomic distribution may reflect historical duplication events, selective retention, or lineage-specific expansion during the evolution of ABA receptor genes [20]. Similar patterns of gene family expansion have been observed in other plant species, suggesting that duplication and subsequent diversification contribute to the functional complexity of *PYL*-mediated ABA signaling networks [15]. Furthermore, the variation in exon–intron organization among *SlPYL* members indicates distinct evolutionary trajectories within different subgroups [27]. The presence of intronless genes and genes containing conserved intron structures may influence transcriptional regulation and contribute to functional specialization among SlPYL proteins.

Promoter analysis revealed that *SlPYL* genes are enriched with diverse *cis*-acting regulatory elements, including light-responsive, hormone-responsive, and stress-related elements (Figure 5). Notably, the presence of abundant ABA-responsive elements (ABREs) suggests that *SlPYL* genes are likely subject to transcriptional regulation by ABA. Similar promoter architectures have been reported in other crops such as eggplants, pears, potatoes, sweet potatoes, and rice [31,33,34,35], supporting the conserved regulatory role of *PYL* genes across plant species. The diverse distribution of stress-, hormone-, and light-responsive *cis*-elements further suggests that *SlPYL* genes are regulated through complex transcriptional networks. This regulatory complexity may contribute to the functional diversification of individual *SlPYL* members under different physiological conditions.

Post-translational modifications (PTMs) represent an important mechanism for regulating protein activity and signaling pathways. In the present study, multiple PTM sites, including phosphorylation, glycosylation, ubiquitination, and acetylation, were predicted in *SlPYL* proteins. Among these, phosphorylation sites were the most abundant (Figure 7), suggesting that phosphorylation may play a major role in modulating SlPYL protein function. Previous studies have shown that PYL proteins can be phosphorylated by CBL-CIPK complexes, influencing ABA signaling and stress responses [36,37,38]. The presence of additional predicted PTMs suggests that SlPYL proteins may have the potential to undergo multilayered post-translational regulation. However, these predictions are based on computational analyses, and experimental validation will be necessary to confirm the occurrence of these modifications and determine their biological significance in ABA signaling.

ABA signaling is closely associated with plant adaptation to environmental stresses, and increasing evidence indicates that ABA-responsive regulatory networks interact with ROS homeostasis and antioxidant defense. Under drought, salinity, and temperature stress, excessive ROS accumulation can cause oxidative damage, whereas antioxidant enzymes such as SOD, POD, and CAT contribute to ROS detoxification and maintenance of cellular redox balance. Functional studies of stress-responsive transcription factors provide experimental support for this relationship. For example, overexpression of *FvNAC29* enhanced salt and cold tolerance in *Arabidopsis* and was associated with increased antioxidant enzyme activities and reduced oxidative damage [9]. Similarly, overexpression of *VhMYB15* increased salinity and drought tolerance and was accompanied by enhanced antioxidant capacity and altered expression of ABA-associated genes [10]. These findings highlight the broader importance of coordinated ABA, ROS, and antioxidant responses in plant stress adaptation. Although the present study did not directly measure ROS accumulation or antioxidant enzyme activity, the stress-responsive cis-regulatory elements identified in the *SlPYL* promoters suggest that individual PYL receptors may be integrated into broader stress-response networks.

The expression profiling revealed that *SlPYL* genes exhibit tissue-specific expression patterns across root, leaf, and bud tissues. In particular, *SlPYL4*, *SlPYL5*, *SlPYL6*, *SlPYL7,* and *SlPYL8* showed relatively higher expression in leaves, while their expression was recorded as relatively lower in plant roots and buds. Notably, all *SlPYLs* exhibited significant expression in plant leaves (except *SlPYL1*), while the expression of *SlPYL3*, *SlPYL4*, *SlPYL5*, *SlPYL6*, and *SlPYL7* was significantly higher in bud tissues as compared to roots (Figure 8A). These findings are consistent with previous studies in other plant species, such as eggplants [33], *B. napus* [15], and pears [34], where *PYL* genes also displayed significant expression patterns in roots, leaves, and bud tissues, respectively. Furthermore, ABA treatment significantly induced the expression of several *SlPYL* genes, particularly *SlPYL3* and *SlPYL7*, across multiple tissues. The induction was more pronounced in leaf tissues compared to roots, while bud tissues also exhibited strong and time-dependent increases in gene expression (Figure 8B). These expression patterns are consistent with the enrichment of ABREs in *SlPYL* promoters, supporting their transcriptional responsiveness to ABA (Figure 5).

Importantly, the enhanced and sustained expression of specific *SlPYL* genes in three respective plant tissues, including leaf, root, and bud tissues following ABA treatment, suggests their potential involvement in ABA-mediated regulation of leaf, root, and bud development. The relative expression level of *SlPYL3* has been noticed as significant (up to ~5-fold) in bud tissues after ABA treatment at 24 hpt, indicating its role in bud growth and development [39]. However, the expression of this gene was recorded as relatively lower in roots and leaves, ~3- and ~4-fold, respectively. Considering the established role of ABA in suppressing bud outgrowth, the observed expression patterns provide indirect evidence supporting the involvement of *SlPYL* genes in regulating the plant architecture of tomatoes. However, these conclusions are based on expression and bioinformatic analyses, and further functional studies are required to elucidate the precise roles of *SlPYL* genes in bud outgrowth regulation.

Overall, this study provides a comprehensive characterization of the *SlPYL* gene family in tomatoes by integrating evolutionary, structural, regulatory, and expression analyses. The conserved protein features, diverse promoter architectures, predicted regulatory modifications, and ABA-responsive expression patterns collectively indicate that *SlPYL* genes represent important components of ABA signaling networks. These findings provide valuable genetic resources and candidate genes for future functional investigations aimed at understanding ABA-mediated regulation of plant architecture and improving crop productivity.

## 4. Materials and Methods

### 4.1. Identification and Characterization of SlPYL Genes

To identify PYL homologs, *A. thaliana* PYL protein sequences were employed as queries in BLASTP searches against fully sequenced green plant genomes available in the BRAD database (http://www.brassicadb.cn/, accessed on 31 December 2025). Retrieved homologous sequences were aligned with AtPYL proteins using ClustalW (https://www.genome.jp/tools-bin/clustalw, accessed on 9 January 2026) under default settings and visualized with GeneDoc (v2.7) [40].

### 4.2. Phylogenetic Relationship Among Arabidopsis, B. napus and Tomatoes

*AtPYL* and *S. lycopersicum* PYL sequences were aligned using the ClustalW module in MEGA X (v10.2.2), and a phylogenetic tree was constructed via the maximum likelihood (ML) approach with 1000 bootstrap replicates and a site coverage cutoff of 95% to account for alignment gaps [41]. *S. lycopersicum* PYL family members were subsequently annotated according to sequence similarity and their clustering patterns relative to AtPYL proteins in the phylogenetic analysis.

### 4.3. Domain, Motif, Gene Structure and Location Prediction Analysis

To characterize the structural features of *S. lycopersicum* PYL proteins, conserved domains were identified using the NCBI conserved domain database (CDD) with an E-value threshold of 1 × 10^−5^. Conserved motifs were detected using the MEME Suite [42] (https://meme-suite.org/meme/, accessed on 20 January 2026), with parameters set to identify a maximum of 5 motifs, motif widths ranging from 6 to 50 amino acids, and allowing only one occurrence per sequence. Gene structure organization, including exon–intron distribution, was analyzed by aligning coding sequences with their corresponding genomic sequences using the Gene Structure Display Server (GSDS; https://gsds.gao-lab.org/Gsds_help.php, accessed on 15 January 2026) [43]. Subcellular localization of PYL proteins was predicted using DeepLoc 2.0 (https://services.healthtech.dtu.dk/services/DeepLoc-2.0/, accessed on 26 August 2026) [44], providing insights into their potential functional distribution within cellular compartments.

### 4.4. Prediction of Cis-Acting Regulatory Elements (CREs)

The promoter sequences were extracted as approximately 2 kb upstream of the annotated transcription start site (TSS) of each *SlPYL* gene based on the NCBI genome annotation. These upstream regions were subsequently analyzed for the presence of putative *cis*-acting regulatory elements (CREs) using the Plantcare database (https://bioinformatics.psb.ugent.be/webtools/plantcare/html/, accessed on 29 January 2026) [45], with particular attention given to elements related to hormonal signaling and stress-responsive regulation.

### 4.5. Protein Tertiary Structure Prediction for BnPYLs

Tertiary structures of SlPYL proteins were predicted using the Phyre2 (Protein Homology/Analogy Recognition Engine) server (https://www.sbg.bio.ic.ac.uk/phyre2, accessed on 4 February 2026) [46]. Protein models were generated based on homology modeling, and the most reliable structures were selected according to confidence scores and sequence coverage.

### 4.6. PTM Sites Prediction Analysis

To investigate potential post-translational regulatory mechanisms, various post-translational modifications (PTMs) in SlPYL proteins were predicted using multiple computational tools, including phosphorylation sites at Ser, as well as glycosylation, ubiquitination, and acetylation sites at lysine (K) residues. Putative palmitoylation, hydroxylation, and methylation sites were predicted at cysteine, prolein and arginine, respectively. To find these potential PTM sites in SlPYLs, an online tool, MusiteDeep (https://www.musite.net, accessed on 8 February 2026) [47] was used. These predicted modifications provide insights into the possible regulatory roles and functional dynamics of SlPYL proteins under varying conditions.

### 4.7. Plant Materials, Growth Conditions, and ABA Treatments

The seeds of *S. lycopersicum* cv Alisa Craig (AC) wild-type (WT) background were obtained from the Laboratory of Germplasm Innovation and Molecular Breeding, Institute of Vegetable Science, Zhejiang University. Seeds were sown in pots containing a 7:3 mixture of horticultural-grade peat moss (Klasmann-Deilmann, Germany) and horticultural-grade vermiculite (commercial grade, Hangzhou, China) and watered with Hoagland’s nutrient solution prepared according to the standard formulation described by Hoagland and Arnon [48]. Conventional growth conditions were maintained as follows: a 14 h day and 10 h night photoperiod, with ambient air temperatures of 25 °C during the day and 20 °C at night, and a photosynthetic photon flux density of 400 μmol m^−2^ s^−1^ [49]. Approximately three-week-old tomato seedlings were used for foliar abscisic acid (ABA) treatments (Sigma-Aldrich, St. Louis, MO, USA; ≥98% purity). The tomato seedlings were sprayed with 50 µM ABA or distilled water (as a control). Environmental conditions in the growth chamber were maintained consistently as described above. The samples were pooled and collected at different time intervals, with each sample being from nine individual plants, immediately frozen in liquid nitrogen and stored at −80 °C for further analyses.

### 4.8. RNA Extraction and Gene Expression Analysis

Total RNA was extracted using TRIzol reagent (Vazyme, Nanjing, China) according to the manufacturer’s protocol. Quantitative real-time PCR (qRT-PCR) was performed using a StepOne Real-Time PCR system (Applied Biosystems, Waltham, MA, USA) with SYBR Green PCR Master Mix (TaKaRa, Dalian, China). Relative transcript levels were calculated using the comparative Ct (2^−ΔΔCt^) method [50]. Gene expression values were normalized against the internal reference gene *SlActin* (*Solyc03g078400*) [51]. Ct values of target genes were first normalized against *SlActin* to obtain ΔCt values, after which the designated reference samples were used as calibrators for calculation of relative transcript abundance.

For tissue-specific expression analysis, the mean ΔCt value of the reference root samples was used as the calibrator and assigned a relative expression value of 1. Relative expression levels of independent experimental root, leaf, and bud samples were calculated relative to this reference root using the 2^−ΔΔCt^ method [52]. For ABA treatment experiments, the mean ΔCt value of the corresponding mock (water-treated) biological replicates at each time point was used as the reference for normalization of ABA-induced expression changes. Thus, ABA-treated samples collected at 6, 12, and 24 hpt were normalized against the corresponding mock samples collected at the same time point (ABA_6 h vs. Mock_6 h, ABA_12 h vs. Mock_12 h, and ABA_24 h vs. Mock_24 h). Relative expression values were calculated from three independent biological replicates and are presented as mean ± SD. Primer sequences used in this study are listed in Table S3.

### 4.9. Statistical Analysis

All experiments were performed using three independent biological replicates, and data are presented as mean ± SD. For qRT-PCR analyses, statistical comparisons were performed using replicate-level ΔCt values rather than the corresponding 2^−ΔΔCt^ values used for graphical presentation [53]. For tissue-specific expression analysis (Figure 8A), ΔCt values of the independent experimental root, leaf, and bud samples were compared with those of the reference root sample. For ABA-responsive expression analysis (Figure 8B–D), ΔCt values of ABA-treated samples were compared with those of the corresponding mock-treated controls at the same time point. Statistical differences between groups were evaluated using Student’s *t*-test in GraphPad Prism v8.0 [54]. Statistical significance was defined as *p* < 0.05. Significant differences are indicated by asterisks (*p* < 0.05, * *p* < 0.01, ** *p* < 0.001), whereas “n.s.” indicates no significant difference.

## 5. Conclusions

In this study, a comprehensive genome-wide analysis of the *PYL* gene family in *S. lycopersicum* identified nine *SlPYL* genes, which were phylogenetically classified into three distinct subfamilies consistent with their homologs in other plant species. The identified SlPYLs exhibited conserved structural features, including the characteristic START domain, conserved motif architecture, chromosomal distribution, and tertiary structures, along with gene structures, which may suggest their conserved roles as ABA receptors. Promoter analysis revealed the enrichment of diverse *cis*-acting regulatory elements, particularly ABA-responsive elements (ABREs), indicating that *SlPYL* genes are likely regulated by hormonal and environmental cues. In addition, the presence of multiple predicted post-translational modification sites indicates the potential for post-translational regulation of SlPYL proteins, although experimental validation is required to confirm these modifications and their biological significance. Exogenous ABA treatment further enhanced the expression of multiple *SlPYL* genes, including *SlPYL3*, *SlPYL7*, *SlPYL6*, and *SlPYL8*, suggesting their potential involvement in ABA-mediated regulatory processes during tomato development. Notably, exogenous ABA treatment significantly induced the expression of *SlPYL3*, with pronounced and sustained expression in buds, and *SlPYL3*, *-4*, *-5*, *-6*, *-7*, and *-8* in leaves. Given the established role of ABA in suppressing bud outgrowth, the ABA-induced expression of *SlPYL* genes in bud tissues may suggest their potential involvement in ABA-mediated regulation of bud outgrowth and plant architecture. Nevertheless, these findings are primarily based on genome-wide computational analyses and expression profiling, and further functional studies are required to validate the predicted roles of *SlPYL* genes.

## Figures and Tables

**Table 1:** Identification of *SlPYL* proteins and their predicted localization.

Gene IDs	Given Names	Chromosomal Position	Start Position	End Position	No. of AA	CDS (bp)	STRAND	Localization
Solyc06g061180.1	SlPYL1	Chr06	391,84,053	391,84,694	213	639	+	Cytosol
Solyc08g076960.1	SlPYL2	Chr08	609,02,761	609,02,066	231	693	−	Cytosol
Solyc12g095970.1	SlPYL3	Chr12	650,70,101	650,68,897	190	570	−	Cytosol
Solyc06g050500.2	SlPYL4	Chr06	331,76,398	331,75,324	275	825	−	Chloroplast
Solyc09g015380.1	SlPYL5	Chr09	871,13,13	871,07,08	201	603	−	Chloroplast
Solyc10g085310.1	SlPYL6	Chr10	645,33,746	645,34,387	213	639	+	Cytosol
Solyc10g076410.1	SlPYL7	Chr10	593,90,084	593,89,473	203	609	−	Chloroplast
Solyc02g076770.1	SlPYL8	Chr02	418,71,601	418,70,945	218	654	−	Cytosol
Solyc06g065410.2	SlPYL9	Chr06	408,30,730	408,32,922	322	966	+	Nucleus

Abbreviations: *SlPYL*, *S. lycopersicum pyrabactin resistance 1-like* genes; Chr, chromosome; AAs, amino acids; CDS, coding sequences.

## Data Availability

The original contributions presented in this study are included in the article/Supplementary Materials. Further inquiries can be directed to the corresponding authors.

## Supplementary Materials

The following supporting information can be downloaded at https://www.mdpi.com/article/10.3390/ijms27177737/s1.

## Abbreviations

The following abbreviations are used in this manuscript:

ABA	Abscisic acid
ABREs	ABA-responsive elements
AC	Alisa Craig
CREs	*Cis*-acting regulatory elements
CDD	Conserved domain database
Chr	Chromosome
GSDS	Gene Structure Display Server
PTM	Post-translational modification
PYL	Pyrabactin resistance 1-like
*SlPYL*	*Solanum lycopersicum pyrabactin resistance 1-like*
