# Supplementary material for: Genome-Wide Analysis of the PYL Gene Family and Its Expression Dynamics in Response to Abscisic Acid in Tomato
Source: Int J Mol Sci. 2026 Aug 29;27(17):7737. doi: 10.3390/ijms27177737 (PMC13566295; doi:10.3390/ijms27177737)
Supplement: Supplementary file 1 [file ijms-27-07737-s001.zip › Supplementary Figure.pdf]

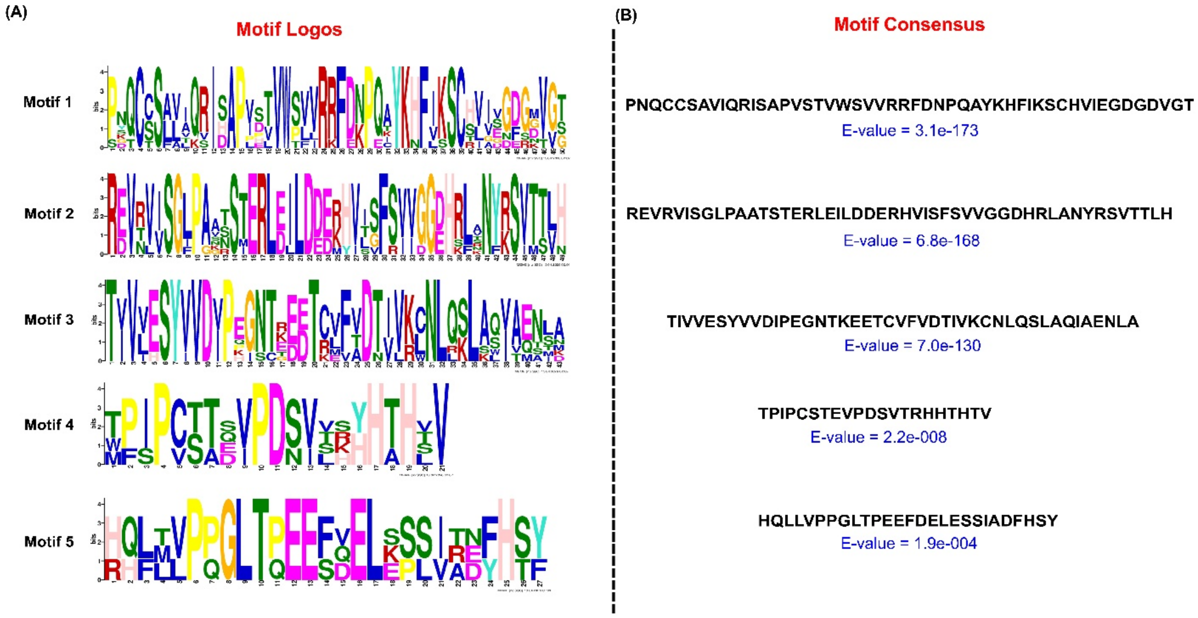

Figure S1. Motif logos and their respective motif consenses with their respective *E*-value.
